# Polar accumulation of pyoverdin and exit from stationary phase

**DOI:** 10.1093/femsml/uqae001

**Published:** 2024-02-14

**Authors:** Clara Moreno-Fenoll, Maxime Ardré, Paul B Rainey

**Affiliations:** Laboratory of Biophysics and Evolution, CBI, ESPCI Paris, Université PSL, CNRS, 75005 Paris, France; Department of Microbial Population Biology, Max Planck Institute for Evolutionary Biology, 24306 Plön, Germany; Laboratory of Biophysics and Evolution, CBI, ESPCI Paris, Université PSL, CNRS, 75005 Paris, France; Laboratory of Biophysics and Evolution, CBI, ESPCI Paris, Université PSL, CNRS, 75005 Paris, France; Department of Microbial Population Biology, Max Planck Institute for Evolutionary Biology, 24306 Plön, Germany

**Keywords:** *Pseudomonas*, pyoverdin, accumulation, polarization, imaging, iron

## Abstract

Pyoverdin is a water-soluble metal-chelator synthesized by members of the genus *Pseudomonas* and used for the acquisition of insoluble ferric iron. Although freely diffusible in aqueous environments, preferential dissemination of pyoverdin among adjacent cells, fine-tuning of intracellular siderophore concentrations, and fitness advantages to pyoverdin-producing versus nonproducing cells, indicate control of location and release. Here, using time-lapse fluorescence microscopy to track single cells in growing microcolonies of *Pseudomonas fluorescens* SBW25, we show accumulation of pyoverdin at cell poles. Accumulation occurs on cessation of cell growth, is achieved by cross-feeding in pyoverdin-nonproducing mutants and is reversible. Moreover, accumulation coincides with localization of a fluorescent periplasmic reporter, suggesting that pyoverdin accumulation at cell poles is part of the general cellular response to starvation. Compatible with this conclusion is absence of non-accumulating phenotypes in a range of pyoverdin mutants. Analysis of the performance of pyoverdin-producing and nonproducing cells under conditions promoting polar accumulation shows an advantage to accumulation on resumption of growth after stress. Examination of pyoverdin polar accumulation in a multispecies community and in a range of laboratory and natural species of *Pseudomonas*, including *P. aeruginosa* PAO1 and *P. putida* KT2440, confirms that the phenotype is characteristic of *Pseudomonas*.

## Teaser

The iron binding compound pyoverdin (fluorescent foci in the image) accumulates at the cell pole in space created by shrinkage of the cytoplasm, and is connected to an advantage in resumption of growth after starvation.

## Significance

Bacteria secrete extracellular products that enable nutrients to be obtained from the environment. A secreted product of relevance for medicine, agriculture, and biotechnology is the iron-chelating siderophore, pyoverdin, which is produced by members of the genus *Pseudomonas*. By analyzing the behavior of single cells we show that on cessation of cell division, pyoverdin localizes to cell poles, but is then released to the environment prior to resumption of cell growth. Of particular significance is the ecological relevance of this behavior: cells that accumulate the siderophore resume growth with minimal delay. Our study reveals a hitherto unrecognized dimension to the biology of Pseudomonas that may prove central to understanding the broader ecological and physiological relevance of pyoverdin.

## Introduction

Extracellular secreted products perform important functions in microbial populations and communities. They provide structure and protection (Flemming et al. 2016), enable coordinated action (Diard et al. 2013, Newman et al. 2017) and also allow acquisition of recalcitrant nutrients, such as polymers that are too large to be internalized (Gilkes et al. 1991), or are otherwise unavailable. An example of the latter is pyoverdin.

Pyoverdin is a naturally fluorescent iron-scavenging chelator (sideropohore) produced by members of the genus *Pseudomonas* (Meyer and Abdallah 1978). Iron is an essential micronutrient that exists in an insoluble state (ferric) in aerobic environments (Neilands 1981). In response to intracellular iron scarcity, pyoverdin biosynthesis begins in the cytoplasm via nonribosomal peptide synthesis and undergoes maturation in the periplasm (Ringel and Brüser 2018). After secretion, it binds Fe^3+^ with high affinity (Ka = 10^32^ M^−1^ for PVDI produced by *P. aeruginosa* PAO1 (Albrecht-Gary et al. 1994)). Once bound to ferric iron the ferripyoverdin complex loses its fluorescent properties, but is recognized by a specific receptor (FpvA) and imported back into the periplasm, where iron is extracted. The pyoverdin molecule is then recycled and can undergo further cycles of export and import (Bonneau et al. 2020).

Because pyoverdin is a soluble extracellular product, it has been widely assumed to be equally available to all members of a community (West et al. 2007, Buckling et al. 2007, Rainey et al. 2014). However, recent work shows that its distribution is subject to cell-level control. In one study pyoverdin producers retained an environment-dependent fitness advantage in conditions where invasion by nonproducers was expected. In light of these experimental results, the possibility of personalization was raised (Zhang and Rainey 2013). Other studies have further supported this notion. For example, in growing microcolonies, pyoverdin diffuses primarily through adjacent cells, reducing loss into the environment (Julou et al. 2013). Furthermore, *Pseudomonas aeruginosa* cells tune periplasmic concentrations of pyoverdin in order to protect against oxidative stress (Jin et al. 2018).

Here, we use time-lapse fluorescence microscopy to study the relationship between *P. fluorescens* SBW25 cells and pyoverdin. Recognizing the importance of spatial structure and contributions therefrom to µm-scale features of microbial assemblages (Hansen et al. 2007, Cordero and Datta 2016, Co et al. 2020, Hartmann et al. 2019), cells were grown on thin layers of agarose set on top of microscope slides. In actively dividing cells, naturally fluorescent apo-pyoverdin is evenly distributed in the periplasm, however, on cessation of growth we observed pyoverdin to accumulate at cell poles. This surprising discovery motivated quantitative analysis, revealing the process of accumulation to be dynamic, reversible, and ecologically relevant.

## Materials and methods

### Strains, culture media, and reagents

Ancestral *Pseudomonas fluorescens* SBW25 originally isolated on beet roots at the University of Oxford farm (Wytham, Oxford, U.K.) (Silby et al. 2009) and a collection of relevant mutants were used: the pyoverdin nonproducer *pvdsG229A*(D77N) named Pvd^-^ in the main text (construction described in (Zhang and Rainey 2013)), the corresponding strain with mCherry fluorescence tagging under IPTG induction, *ΔmreB*, PBR716 (Δ*wsp*Δ*aws*Δ*mws*, described in (McDonald et al. 2009)). Pyoverdin import and export defective mutants *ΔPFLU3979* (OpmQ) and *ΔPFLU2545* (FpvA) were created by two-step allelic exchange (Zhang and Rainey 2007a). The periplasm reporter strain was created by fusing the red fluorescent protein mScarlet to the signal peptide of periplasmic protein, DsbA (see Supplementary Methods). A neutrally marked SBW25 strain (Zhang and Rainey 2007b) was used to replicate the fitness assays from (Zhang and Rainey 2013). *Escherichia coli* DH5α λ_pir_ and pRK2013 were used for cloning. *Bacillus* 002.IH from Steven Quistad’s compost heap collection (Quistad et al. 2020) was used for the community experiment. For the phylogenetic comparison, an assortment of species of the genus *Pseudomonas* were selected from the lab collection. Most experiments were performed in succinate minimal medium (SMM, described in (Julou et al. 2013)). Overnight cultures were done in Luria-Bertani (LB) broth. Replication of fitness assays was performed in CAA and KB media as described in the original work (Zhang and Rainey 2013). Community experiment was done in M9 minimal medium with cellulose as the only carbon source (Whatman). Where indicated media was supplemented with 2,2′-dipyridil (Sigma), tetracycline (Duchefa, France), Fe_2_[SO_4_]_3_(III) (Sigma), IPTG (Melford). For strain construction tetracycline, nitrofurantoin (Sigma), X-gal (Melford), D-cycloserine (Duchefa) were used.

### Agarose pad

To prepare the agarose pad, 220 µL of agarose (Melford) dissolved in SMM (2$\%$ w/v) were poured onto a microscope slide fitted with a sticky frame (Gene Frame, Fisher Scientific), pressed with a clean slide and allowed to dry for ∼ 2 min. A small ∼ 3 mm section of the pad and frame was cut across the slide to provide air for the growing cells. 1.5 µL of washed culture was inoculated on the agarose pad and sealed with a coverslip.

### Microscopy

Inoculated agarose pads were monitored by taking snapshots every 30 min for a typical total time of 18h using the microscope Axio Observer.Z1 (Zeiss, Germany). Cells were imaged under phase contrast (exposure: 100 ms) and fluorescence corresponding to Pvd using the fluorescence Source X-Cite 120 LED and the following filters: 390/40 BrightLine HC, Beamsplitter T 425 LPXR, 475/50 BrightLine HC (exposure: 30 ms, 12 $\%$ intensity). Images were taken with 63x and optovar 1.6x magnification.

### Image analysis

Image processing was performed using the image analysis software Image J. Segmentation and analysis was performed using the package for Matlab SuperSegger from the Wiggins Lab (Stylianidou et al. 2016). Further analysis and data visualization was carried out with the programming software R. After segmentation cells were sorted by polarization status using a classificator obtained with the Statistics and Machine Learning Toolbox for Matlab (More information in Supplementary Methods).

### Community experiment

SBW25 and *Bacillus* 002.IH (from (Quistad et al. 2020)) were grown on 20 ml M9 minimal medium and cellulose as the only carbon source (1 cm x 1 cm cellulose paper). After overnight growth cultures were washed and co-inoculated into 20 ml of M9 medium with cellulose paper in a 60 ml vial. These vials were incubated at room temperature with unscrewed caps to allow air exchange, and periodically sampled for imaging.

### Phylogenetic comparison

A collection of laboratory and natural strains belonging to the genus *Pseudomonas* were subjected to a binary qualitative polarization test, i.e. the test was considered positive if polarized cells were observed under fluorescence microscopy in conditions similar to SBW25 but no dynamics were assessed. Commonly used laboratory strains *P. aeruginosa* PAO1 and *P. putida* KT2440 were tested. Natural isolates belong to collections from two different locations. Paris, France (Quistad et al. 2020): T24 V1a, T24 V9b, T24 H1b, T24H9b; all classified as *P. putida*. Oxford, UK (Zhang et al. 2020): *P. marginalis* U106, *P. putida* U177, *P.aureofaciens* U149, U180, and U181.

## Results

**Figure 1.**
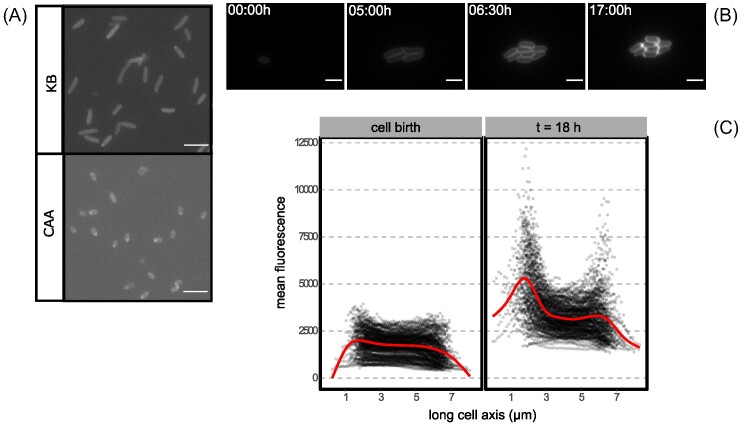
Accumulation of pyoverdin in *P. fluorescens* SBW25 at the cell pole. (A) Snapshots of experiments described in (Zhang and Rainey 2013) where fitness assays of pyoverdin producing SBW25 and a nonproducing *pvdS* defective mutant yield contrasting results depending on the culture medium. In both cases the environment is unstructured and ancestral SBW25 producer cells are rare, inoculated at  1%. A fitness advantage to nonproducing cells in KB was previously reported, but the reverse in CAA (Zhang and Rainey 2013). In KB (top) pyoverdin nonproducing cells rarely showed evidence of accumulation of pyoverdin, whereas (bottom) this was common in CAA cultured cells where accumulation is visible at the pole. Images were obtained from 3 µl samples of these experiments imaged under fluorescence light to visualize the distribution of pyoverdin. All scale bars correspond to 10 µm. (B) Fluorescence time-lapse images of a growing microcolony of SBW25 in a SMM agarose pad. Images represent selected time points including, respectively: the initial inoculum, exponential growth, end of exponential growth (i.e., the final number of cells in the colony) and end of time-lapse acquisition (18 h total). (C) Mean fluorescence intensity along the long axis of cells in a growing microcolony, when the last generation of cells is born (left) and at the end of acquisition (t = 18 h, right). Black dotted line represents the fluorescence profile of individual cells, the red line represents a smoothed mean of all the cells. Between 0 h and 18 h accumulation of pyoverdin is evident, especially at the pole.

*Pseudomonas fluorescens* SBW25 (hereafter SBW25) is a model bacterium (Silby et al. 2009) known to produce pyoverdin (Moon et al. 2008) and other secreted products (Hammerschmidt et al. 2014, Spiers et al. 2002). In a previous study Zhang & Rainey (Zhang and Rainey 2013) provided evidence of pyoverdin personalization that was inferred following contrasting outcomes of fitness assays performed under different conditions. We reproduced these assays, but rather than examining frequencies of producers and nonproducers by plating, aliquots were observed by fluorescence microscopy. In casamino acids medium (CAA), the medium where an unexpected advantage for pyoverdin producers had been described, cells exhibited internal pyoverdin accumulation more evident at the pole where fluorescent foci (Fig. 1A) appeared. This initial observation provoked further analysis.

To accurately characterize subcellular patterns of pyoverdin, time-lapse fluorescence images of ancestral SBW25 were obtained in defined succinate minimal medium (SMM), where succinate acts both as a carbon source and a weak iron chelator. During exponential phase and early stationary phase, SBW25 cells showed a phenotype typical of fluorescent *Pseudomonas* with pyoverdin being homogeneously distributed in the periplasm, while in late stationary phase fluorescent pyoverdin foci appeared at the cell pole (Fig. 1B). Superposing cell fluorescence profiles obtained by image segmentation confirmed that both polar localization and accumulation of the siderophore occurred at this later stage (Fig. 1C).

**Figure 2.**
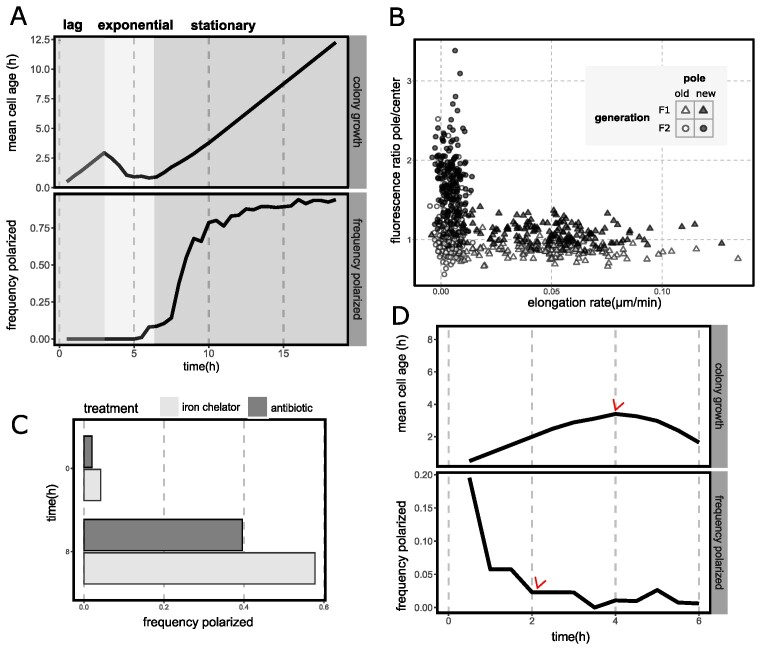
Polar accumulation is a reversible phenotype associated with arrest of cell division. (A) Polarization in different growth stages of a microcolony. Mean age of the cells in a growing microcolony of SBW25 (black line, top) and the corresponding frequency of polarized cells for each time point (black line, bottom). Colored panels represent the growth stages of the microcolony, from left to right: lag phase (generation F0), exponential phase (generation F1), and stationary phase (generation F2). N = 35, 159, 187, respectively. In all cases data has been filtered to exclude cells with segmentation errors or other artifacts that preclude proper analysis. Note that as microcolonies begin to form in exponential phase and cells are no longer isolated, overlap between adjacent cells creates regions of high fluorescence that could lead to classification errors. Nevertheless, visual inspection reveals that cells remain in a homogeneous state during exponential growth, with polarization onset being clearly associated to stationary phase. To maintain a consistent physiological response, in this study cells are manipulated from a starting point corresponding to early stationary phase (Fig. S4). Elongation rate and accumulation of fluorescence at the cell pole of individual cells in a growing microcolony. Data extracted from (B), markers represent individual cells in different growth phases of the colony (F1, exponential, triangle markers; F2, stationary, circle markers) and the old (white markers) and new (black markers) cell pole. Elongation rate is represented by the average over the lifetime of a cell. Accumulation of fluorescence at the pole is represented by the maximum ratio over the lifetime of a cell of the sum of the pixels in the pole region and central region of a cell. These regions are defined by segmenting the cell and dividing it in 3 portions over the long axis, where the external 1/3 represent each pole and the remaining 1/3 represents the center. (C) Polarization in response to chemical stresses related and unrelated to iron metabolism. Bars represent the frequency of polarized cells at the start of treatment and after 8h of treatment with either 100 µg/ml 2,2′-dipyridil (DP) (light bars) or 5 µg/ml tetracycline (dark bars). No cell division was observed during treatment. N = 99 (t = 0 h), 94 (t = 8 h) and N = 94 (t = 0 h), 79 (t = 8 h) for DP and tetracyline treatments respectively. (D) Depolarization and subsequent growth of cells pre-treated with an iron chelator. Plot represents colony growth and polarization as in (A). Cells were treated with 100 µg/ml DP during 4h, washed and inoculated on a fresh SMM agarose pad. Data corresponds to five technical replicates i.e., five positions on the agarose pad. Red arrows indicate the time point where the colony overall starts growing (top) and where the majority of the cells are depolarized (bottom). Total initial number of cells N = 87.

By examining cell division throughout the time-lapse series it is possible to explore the relationship between time and population growth. The corresponding frequency of polarized cells (Fig. 2A) was tracked by classifying segmented cells automatically as “polarized” (accumulated) or “homogeneous” (non-accumulated) using a machine learning algorithm (Supplementary Materials and Methods). After inoculating the microscope slide, cells undergo a period of acclimation to the medium without division. We observed no accumulation events during this phase. As division begins, age of cells in microcolonies decreases until it reaches a minimum that marks exponential phase. Pyoverdin continued to be non-polarized. Note that the small frequency of cells identified as “polarized” in the plot falls within the range of classification error. Finally, the population enters stationary phase and cells continue ageing without division. Accumulation of pyoverdin at the pole increased within the first few hours, encompassing most cells within the population by 18h (Fig. S1). As stationary phase progresses, cells become classified as “polarized” at different rates while possessing varying amounts of intracellular pyoverdin, suggesting that the quantity of internal pyoverdin does not define polar localization per se (Fig. S2A). No significant difference was observed in the total amount of internal pyoverdin between cells classified as “polarized” or “homogeneous” (Fig. S2B), despite the fact that the majority of cells accumulate pyoverdin over time (Fig. 1C).

While the time-averaged state of microcolonies exposes population-level dynamics of polarization – namely, that accumulation happens in stationary phase – it obscures the behavior of individual cells. To specifically analyze single cells, cells were grouped according to division status over three generations: F0 for initial inoculum (cells where birth could not be identified but division was observed), F1 for cells in exponential phase (born from the division of F0 cells and underwent division later) and F2 for the daughter cells of F1 that entered stationary phase and remained constant for the final hours of the experiment. Growth measurements from F1 cells, and pyoverdin accumulation measurements from F2 cells, corroborate – despite some variability in the onset of accumulation – that accumulation is incompatible with active cell division. Cells either elongate or accumulate fluorescence (pyoverdin) at the cell pole. This result holds for both the old and new pole (Fig. 2B). Curiously, pyoverdin accumulates preferentially at the new pole, but not exclusively, and sometimes distinct foci are present at both (Fig. S3)

Stationary phase marks cessation of cell division due to nutrient depletion. On an agarose pad, entry into stationary phase is highly variable, with access of cells to nutrients and oxygen depending on position and proximity to neighbouring cells. To study pyoverdin accumulation under controlled conditions cells were exposed to two stressors that abruptly arrest cell division, but by different mechanisms: 1, an iron chelating agent (2,2′-dipyridil (DP)) and; 2, a protein synthesis-disrupting antibiotic (tetracycline) (Fig. S5A). Both stressors induced accumulation at the pole (Fig. 2C).

Precisely because polarization appears when cells are starved and/or stressed, processes associated with cell death, such as cell wall damage, are potential elicitors. We thus asked whether pyoverdin accumulation was connected to cell viability. Bacteria were treated with DP, which reliably induces polarization (Fig. 2C) and then transferred to a fresh agarose pad. Time-lapse imaging revealed that polarization is reversible and that depolarization precedes exit from lag phase (Fig. 2D). Polarized cells consistently re-established a homogeneous pyoverdin distribution within the first few hours after inoculation, with elongation and division resuming at a later time point (Fig. S5B). Note that both the initial frequency of polarized cells and the time to depolarization were counted from the start of image acquisition. During the previous experimental manipulation, some individuals might have altered their polarization status. Despite this, the trend is clear, with depolarization occurring before resumption of growth. Polarization is thus not a consequence of cell death but rather a reversible, dynamic process tied to arrest of cell division.

**Figure 3.**
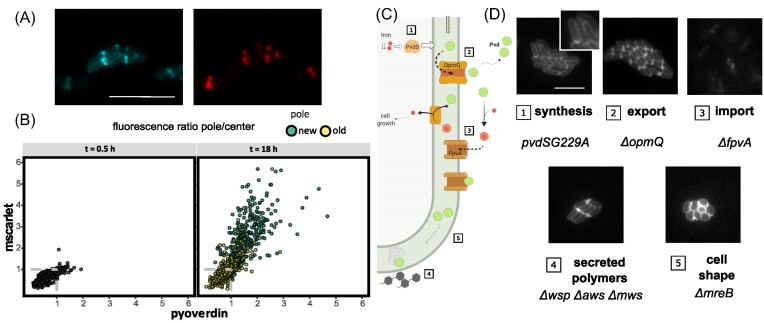
Untangling the mechanism of polarization. (A) A recent study (Shi et al. 2021) showed that upon starvation the cytoplasm of *E. coli* cells shrinks creating extra space in the periplasm at one of the cell poles. To check if a similar phenomenon happens in *P. fluorescens* SBW25 we expressed a red fluorescent mScarlet protein fused to the localization signal peptide of dsbA, a periplasmic protein. The image obtained at t = 18 h shows co-localization of mScarlet (bottom, red) and pyoverdin fluorescence (top, blue, scale bar represents 10 µm). (B) Quantitative analysis of time-resolved images at 30 min and 18 h. Cells were segmented and intensity of fluorescence was measured along the long axis. Fluorescence at the poles was compared with the signal produced at the center of each cell; this was done for both pyoverdin (x-axis) and mScarlet (y-axis). Dots represent the intensity ratio for both fluorescent molecules. For measurements at 18 h the values for both the new pole (green dots) and old pole (yellow dots) are represented. Polar information is not available for freshly inoculated cells. The gray dashed line highlights the values where the center of the cell and the poles are not substantially different, i.e., their fluorescence ratio is close to 1. Deviation from this value indicates polar accumulation of the molecules. C) Cartoon depicting a simplified version of the pyoverdin pathway in which candidate genes for polarization are shown. 1. Pyoverdin (Pvd) synthesis starts in the periplasm in response to iron scarcity mediated by the transcription factor PvdS. Pyoverdin is then secreted to the bacterial periplasm, where it matures and becomes fluorescent. 2. Periplasmic pyoverdin is exported into the external medium by a complex that includes the transporter OpmQ. There, it chelates insoluble iron (Fe^3+^). 3. Ferripyoverdin complexes (no longer fluorescent) are then imported back into the periplasm after binding to the receptor FpvA. This receptor is known to also bind free pyoverdin (Ringel and Brüser 2018). In the periplasm, iron is extracted and pyoverdin is again recycled into the external medium by OpmQ. Polarization might also be determined by genes unrelated to the pyoverdin pathway: 4. SBW25 is known to secrete polymers such as cellulose that might trap pyoverdin (Spiers et al. 2002). 5. Pyoverdin could accumulate at the cell poles due to the rod shape of SBW25. (D) Mutants associated to the main processes depicted in (A) and their phenotype with regards to pyoverdin polarization. Mutants were grown on an agarose pad as described and fluorescence images displaying pyoverdin were taken at the time points indicated in the photo. The pyoverdin nonproducing mutant *pvdSG229A*(D77N) was co-inoculated with SBW25 to enable access of the mutant to pyoverdin. Mutants were tagged with a red fluorescent protein, one mutant colony is displayed in the image.

Pyoverdin polarization is clearly connected to the physiological state of cells, but how this is achieved is unclear. A recent study showed that the cytoplasm of *E.coli* shrinks on starvation leading to enlargement of periplasmic space at cell poles (Shi et al. 2021). We hypothesized that this might also occur in SBW25 cells, with the space created by shrinkage allowing accumulation of pyoverdin. To this end, a red fluorescent protein (mScarlet) was translationally fused to the peptide signal from *dsbA* thus targeting mScarlet to the periplasm (Shi et al. 2021). Having confirmed periplasmic localization, cells were grown on agarose pads and the distribution of mScarlet was tracked by microscopy. At inoculation, mScarlet was homogeneously distributed as observed for pyoverdin, however, on entry into stationary phase, the fluorescent marker accumulated at the poles of the cells (Fig. 3A). As with pyoverdin, mScarlet accumulation was biased toward new poles (Fig. 3B). As expected, unlike pyoverdin, mScarlet showed no evidence of secretion to the external medium (Fig. S8).

While overlap in patterns of pyoverdin and periplasm-targeted mScarlet accumulation points towards the fact that pyoverdin polarization is coupled to general cellular phenomena, possibilities remain for the involvement of genetic determinants of pyoverdin biosynthesis, regulation and / or transport. To test this hypothesis, patterns of pyoverdin accumulation were monitored in a range of mutants.

First, polar accumulation of pyoverdin is not a consequence of defective periplasmic maturation (Yeterian et al. 2010): SBW25 producer and nonproducer types (the latter with an mCherry fluorescent marker) were co-cultured. Pyoverdin was localized in nonproducing cells (Fig. 3.1) demonstrating that accumulated pyoverdin is functional, since any siderophore internalized by Pvd^-^ necessarily comes from the external medium after export by SBW25. Polarization thus involves pyoverdin that is actively used among cells of the population.

Next, recognizing that mature pyoverdin interacts with membrane proteins via OpmQ (Yeterian et al. 2009, Schalk et al. 2001), *opmQ* was deleted and the pattern of pyoverdin accumulation determined. SBW25 Δ*opmQ* showed no change in capacity to accumulate pyoverdin at cell poles (Fig. 3.2), however, the mutant showed increased intracellular levels of pyoverdin; secretion was not abolished (Schalk and Guillon 2013) (Fig. S6A).

Pyoverdin synthesis is subject to positive feedback control via the pyoverdin receptor FpvA making this protein a candidate for involvement in pyoverdin accumulation (Ringel and Brüser 2018). Production of pyoverdin by SBW5Δ*fpvA* was significantly reduced compared to the ancestral type (Fig. S6B), however, where pyoverdin was visible (thus accumulated), it was typically polarized as in ancestral SBW25 (Fig. 3.3).

While the genes involved in extraction of ferric iron and translocation into the cytoplasm have not been characterized in *P. fluorescens*, a recent study in *P. aeruginosa* offered potential candidates for polarization including a siderophore-binding periplasmic protein (Bonneau et al. 2020). Deletion of the homologs of these genes in SBW25 PFLU_2048, PFLU_2041, and PFLU_2043 did not disrupt accumulation of pyoverdin (Fig. S7). Thus, neither aggregation of pyoverdin at trafficking points across the periplasm, nor accumulation via the ferripyoverdin-complex recycling, are involved in polarization.

Additional possibilities for polar accumulation are via connections to extracellular polymer synthesis and cell morphology. SBW25 secretes cellulose when constructing bacterial mats at the air-liquid interface (Spiers et al. 2002, Ardré et al. 2019) which could trap pyoverdin at the perimeter of cells. However, pyoverdin localization is not altered in SBW25 Δ*wsp*Δ*aws*Δ*mws* (McDonald et al. 2009) that is unable to produce cellulose (Fig. 3.4). Finally, the rod-cell-shape characteristic of *Pseudomonas* was considered a possible contributory factor. To test this, pyoverdin accumulation was observed in a spherical Δ*mreB* mutant (Yulo et al. 2018). Despite the aberrant cell shape, this mutant displays fluorescence foci of pyoverdin after extended culture (Fig. 3.5). Curiously, pyoverdin accumulation at the pole is also independent of cell size in the ancestral genotype (Fig. S9).

Polar accumulation of pyoverdin is linked to cell physiology, but is unperturbed by known genetic determinants of pyoverdin biosynthesis, regulation, or transport. This leads to the possibility that pyoverdin accumulation under conditions of cellular stress is an accidental consequence of the biophysics of cell biology and lacks ecological relevance. The alternate hypothesis is one of ecological relevance: a plausible explanation being that release of accumulated pyoverdin allows cells to reduce the time required to exit stationary phase.

An ideal test would be an experiment in which time to resumption of growth of an ancestral type that accumulates pyoverdin is compared to a mutant unable to accumulate pyoverdin, under conditions where pyoverdin accumulation both occurs, and doesn’t occur. If accumulation of pyoverdin confers a fitness advantage, then ancestral types are expected to resume growth more rapidly than non-accumulating mutants, but only after cells have first experienced conditions that promote accumulation of pyoverdin.

**Figure 4.**
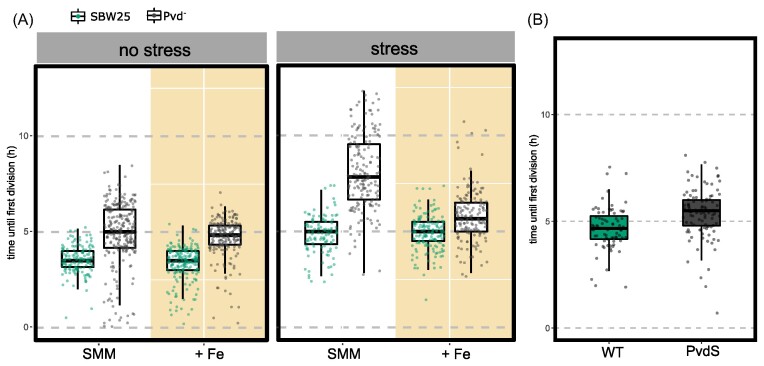
Pyoverdin accumulation facilitates recovery of growth after stress. (A) Time until first division (lag time) of SBW25 (green) and pyoverdin defective mutant *pvdS229*(D77N), termed Pvd^-^ (gray) under different treatments and conditions. Prior to inoculation cells were either grown in the usual culture medium SMM (no stress on left panel) or treated with DP for 4h (stress on right panel). Cells were then inoculated on a fresh agarose pad, supplemented with 0.45 mM Fe_2_[SO_4_]_3_: these are labeled “+ Fe” (yellow background); unsupplemented SMM treatments are labeled “SMM” (white background). Dots represent individual cell values, box plots represent the associated distribution (median, 25th and 75h percentiles) N = 145, 261, 199, 193, 111, 168, 156, 156, respectively, from left to right. (B) Time until first division of SBW25 (green) and Pvd^-^ mutants co-inoculated in a fresh agarose pad after separate stress treatment (4h in DP). Pvd^-^ mutants were labeled with a red fluorescent protein to allow identification of individual cells. Dots and box plots as in (A). N = 175 total cells.

Unfortunately, as evident above, no such pyoverdin-accumulating mutant exists, nonetheless, progress is possible via an experiment that exploits the fact that a PvdS mutant cannot accumulate pyoverdin because it is unable to produce it. To this end, SBW25 and a PvdS mutant (SBW25 *pvdSG229A*(D77N) (Zhang and Rainey 2013) (hereafter termed Pvd^-^) were grown separately in SMM for sufficient time to ensure cells entered stationary phase (24 h). A sample of cells from both populations was then transferred to independent SMM agarose pads and the time to first cell division determined. While a slight delay in median time for resumption of growth was observed in Pvd^-^ compared to SBW25 the distribution of data points shows significant overlap. The findings were not affected by transfer of cells from overnight SMM culture to agarose pads additionally supplemented with excess iron (Fig. 4A, left). This indicates that even the Pvd^-^ mutant was not deficient in iron despite having entered stationary phase.

Results were significantly different when the 24 h pre-culture period in SMM included a final 4 h period during which populations of both SBW25 and Pvd^-^ experienced iron-stress arising from supplementation of SMM with the iron chelating agent DP. As shown above, a 4 h treatment with DP not only caused cessation of growth, but also triggered polarization and accumulation of pyoverdin. The effect of this treatment was to greatly extend the time to resumption of growth in the non-pyoverdin-accumulating mutant. This difference was eliminated when cells, having experienced iron-deprivation, were provided with excess iron (Fig. 4A, right). These results demonstrate a fitness advantage to accumulation of pyoverdin that stems from a reduction in time to exit stationary phase.

This conclusion is further supported by the results of an additional experiment in which SBW25 and the Pvd^-^ mutant were grown as previously, treated with DP and then mixed in a 1:1 ratio and then transferred to a fresh SMM agarose pad. Measurement of the time to resume growth showed little difference among the competing genotypes (Fig. 4B). This is consistent with the expectation that SBW25 releases accumulated pyoverdin into the (fresh) medium to the benefit of both producer and non-producer alike. Notable though is the fact that producer cells reap greatest benefit. When nonproducers are instead inoculated in the presence of rare ancestor cells ($\sim 1\%$) their lag time again increases significantly, supporting the conclusion that newly released pyoverdin underpins the faster division of producers (Fig. S10). Furthermore, the amount of pyoverdin accumulated during starvation seems to be sufficient to support at least twice as many cells, as the lag time of SBW25 producer cells was not affected by the presence of nonproducers.

**Figure 5.**
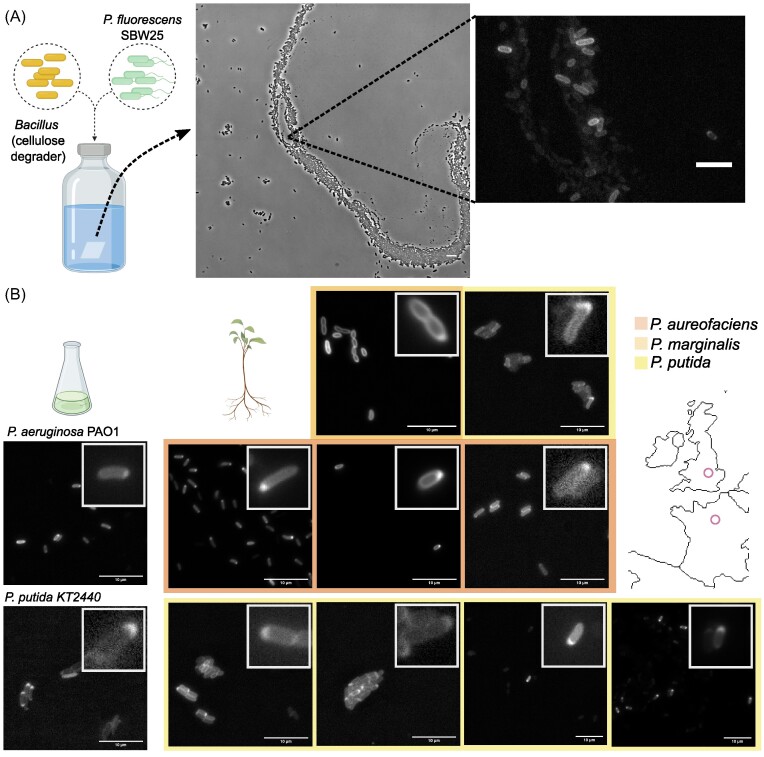
Accumulation visible by polarization is evident in SBW25 in a multispecies community and is a common phenotype in related species of the genus *Pseudomonas*. (A) Polarized SBW25 cells in a multispecies community with interdependencies. SBW25 and a cellulose-degrading *Bacillus* strain isolated from a compost heap in Paris, France (Quistad et al. 2020) were co-cultured in glass vials with minimal medium and cellulose paper as the only carbon source. The community was periodically sampled to assess the polarization state of SBW25. A representative image obtained after 28 days of growth is displayed, where both strains are visible (left, phase contrast image) and in a magnified region where pyoverdin distribution in SBW25 is visualized (right, fluorescence image). (B) Qualitative accumulation assessment in species of *Pseudomonas* other than SBW25. A collection of laboratory (left) and natural (right) strains were tested for accumulation as evident by polarization. Accumulation by polarization in commonly used laboratory strains: *P. aeruginosa* PAO1 tested in SMM agarose pad with 1000 µg/ml DP for 7:30 h; *P. putida* KT2440 tested in SMM agarose pad for 24h. Polarization test in natural isolates, left to right and top to bottom: U106 (liquid KB medium for 24 h); U177 (SMM agarose pad for 24 h); U149 (liquid SMM with DP 100 µg/ml for 24 h); U180 (liquid SMM with DP 100 µg/ml for 24 h); U181 (SMM agarose pad for 16 h); T24 V1a (SMM agarose pad for 24 h); T24 V9b (SMM agarose pad for 24 h); T24 H1b (SMM agarose pad for 24 h); T24 H9b (liquid SMM for 24 h). Natural isolates were collected in Oxford, UK (Zhang et al. 2020) (first and second row) and in Paris, France (Quistad et al. 2020) (Bottom row)(locations are roughly marked with a pink circle on the map). Color represents the identified *Pseudomonas* species. Insets highlight cells with clearly accumulated pyoverdin.

We further tested pyoverdin polarization in conditions closer to the natural milieu of SBW25, where species inter-dependencies are common and resources invariably limiting. To implement a minimal bacterial community, SBW25 was co-cultured with a cellulose-degrading *Bacillus* isolated from a compost heap (Quistad et al. 2020). Because SBW25 is unable to degrade this polymer, it must rely on the *Bacillus* species to obtain carbon for growth. These two strains were grown together in minimal medium with cellulose paper as the sole carbon source and periodically imaged to assess polarization status. Pyoverdin was readily observed to be polarized (Fig. 5A). Pyoverdin accumulation at the cell pole is thus likely to be part of the natural phenotypic repertoire of SBW25, and given that in natural environments bacteria likely find themselves in or close to stationary phase most of the time, polar accumulation of pyoverdin stands to be the normal state.

Since pyoverdins are produced by many species of the genus *Pseudomonas*, investigating subcellular distribution in related strains may provide an evolutionary context for polar accumulation. We selected 11 strains belonging to the genus *Pseudomonas*, comprising both common laboratory strains (such as *P. aeruginosa* PAO1, in which pyoverdin is usually studied) and natural isolates from different European locations. The ability of these strains to polarize pyoverdin was classified qualitatively, that is, presence or absence of polarized cells (Fig. 5B). All tested strains localized pyoverdin at the cell poles in conditions similar to those described here for SBW25. Interestingly, different strains polarized pyoverdin with treatments of varying stringency. In some cases extended culture in SMM was enough to observe the phenotype (e.g., *P. putida* KT2440). In others, amendment with an iron chelator was necessary to observe the effect (e.g., *P. aureofaciens* U149), at the same dosage that induced polarization in SBW25, and in the specific case of *P. aeruginosa* PAO1, very high doses of DP were required. This range of responses could reflect the secretion of secondary siderophores by some strains (Cornelis and Matthijs 2002) or differences in the regulation of pyoverdin production (Rainey et al. 2014), and suggest that polarization is an ecologically relevant trait that varies depending on the evolutionary history of the lineage.

## Discussion

Previous work showing that the population-level distribution of pyoverdin changes depending on nutrient status (Zhang and Rainey 2013), contact with neighboring cells (Julou et al. 2013), and environmental stress (Jin et al. 2018) motivated our investigation. With focus on *P. fluorescens* SBW25, and using time-resolved microscopy, we have shown that pyoverdin transiently accumulates within cells (preferentially at cell poles), that localization is a reversible process associated with arrest of cell division, and is affected by factors such as entry into stationary phase and deprivation of specific nutrients (Fig. 2). Particularly significant is demonstration that accumulation of pyoverdin has ecological relevance (Fig. 4).

Investigations at the level of individual cells allow cell-level behaviors to be linked to the dynamics of populations (Cordero and Datta 2016, Co et al. 2020). For example, Gore et al. (2009) (Gore et al. 2009) showed that the cellular location of invertase responsible for degradation of sucrose in *Saccharomyces cerevisiae* creates diffusion gradients of degradation products that deliver benefit to producing cells, despite costs to producers of synthesizing the enzyme (Gore et al. 2009). Similarly, the siderophore enterochelin (a catecholate secreted by *E. coli* and other *Enterobacteriaceae*) can remain associated with the outer membrane under conditions of low cell density, delivering preferential benefit to enterochelin-producing cells (Scholz and Greenberg 2015). Some localized products display dynamic behaviour (Shapiro et al. 2002), with, in some instances, proteins oscillating between poles (Raskin and De Boer 1999). In other instances, proteins accumulate via formation of nuclear occlusions in the cytoplasm (Laloux and Jacobs-Wagner 2013). In the context of pyoverdin, complexes termed “siderosomes” localize enzymes for synthesis of pyoverdin at old cell poles during exponential growth by association of the cytoplasmic membrane with L-ornithine N5-oxygenase (encoded by *pvdA*) (Guillon et al. 2012, Gasser et al. 2015).

While pyoverdin in SBW25 was most often observed at new cell poles, it was also evident at old poles; on occasion it was found at both poles. Moreover, although all cells accumulate pyoverdin (Fig. 1C), a minority show no evidence of polar localization. Measurement of the amount of pyoverdin in cells that localize the iron chelator showed that it is the same as that contained within cells displaying a homogeneous distribution of pyoverdin (Fig. S2).

Taken together, ambiguity surrounding accumulation and polar localization suggested a general connection to biophysical aspects of cell biology. Interestingly, a recent study of *E. coli* under starvation conditions showed polar accumulation of fluorescent markers, including mCherry (Shi et al. 2021). Accumulation was connected to shrinkage of the cytoplasm, with shrinkage creating an expansion of space in the polar region of the periplasm. It thus seemed plausible that accumulation of pyoverdin in SBW25 in stationary phase may be a consequence of nonspecific cellular behavior under growth arrest. Such a possibility was bolstered by the fact that a PvdS mutant deficient in synthesis of pyoverdin (and thus lacking siderosomes) nonetheless accumulated pyoverdin, that deletion of genetic determinants of uptake and recycling had no observable effects on accumulation, and that pyoverdin accumulation also occurred at discrete locations in spherical Δ*mreB* cells (Fig. 3D.5).

To investigate the hypothesis that pyoverdin accumulation is connected to cytoplasmic shrinkage, red fluorescent mScarlet was translationally fused to the localization signal peptide of DsbA. As cells entered stationary phase accumulation of the fluorescent marker protein was observed at cell poles in a manner analogous to that observed for pyoverdin. In fact, single images captured using filters specific for the reporter protein and pyoverdin showed an almost perfect overlap (3A,B). This lead us to conclude that polar accumulation of pyoverdin is part of a general cellular response to starvation. Thus observations made in *E. coli* with a chimeric reporter (Shi et al. 2021) appear to hold for SBW25, but with our observations connecting cytoplasmic shrinkage to accumulation of a biologically relevant molecule. Additionally we demonstrate that accumulation delivers beneficial effects on fitness.

Data in Fig. 4A show that cells that have accumulated pyoverdin – most of which is localized to cell poles – resume growth more rapidly compared to cells that have not accumulated pyoverdin. A time-to-first cell division advantage was not evident when growth-arrested cells were transferred to iron-replete conditions. This demonstrates that liberation of the stock of pyoverdin accumulated during growth cessation speeds the time to growth resumption after growth arrest, presumably through provision of available iron to growing cells 4B.

Of additional interest is evidence from cell-level observations that rare pyoverdin producers are largely unaffected by the presence of Pvd^-^ mutants (Fig. S10). Such mutants have often been referred to as “cheats” and thus expected to negatively impact the fitness of pyoverdin producers (so called “cooperators”). Lack of detrimental impact is further evidence that pyoverdin production preferentially benefits producer cells. This finding further calls into question the fit between social evolution theory and production of extracellular products by microbes (Zhang and Rainey 2013, Rainey et al. 2014). It also gels with work showing that populations of siderophore-producing *Pseudomonas* can be grown in the presence of an excess of nonproducing mutants without significant reduction in overall yield (Özkaya et al. 2018).

Recent work suggests important physiological differences between acute (and unexpected) interruptions of cell division, and gradual growth arrest that determines entry into stationary phase (Kaplan et al. 2021). In the former case, sudden arrest induces a disrupted state that results in large variability in cell-level duration of lag phase. In the latter case, cells implement genetic programs that buffer the effects of impending starvation creating cohesive population-level responses (Kaplan et al. 2021). The seemingly adaptive nature of pyoverdin accumulation under stress is reminiscent of – and perhaps even connected to – the capacity of SBW25 to enter a semi-quiescent capsulated state upon starvation. During starvation, SBW25 cells produce an excess of ribosomes that allow rapid exit from stationary phase once growth-permissive conditions are encountered. Cells unable to provision ribosomes are quickly out-competed by those that do (Remigi et al. 2019). It is possible that localization of pyoverdin, followed by fast release under growth permissive conditions, has evolved as a strategy precisely because it maximizes competitive performance upon resumption of growth. Rapid resumption of growth is likely to deliver significant fitness benefits in environments punctuated by periods of nutrient abundance and scarcity.

Mounting evidence indicates that pyoverdin – in its many variant forms (Cornelis and Matthijs 2002) – has multiple impacts on composition and function of microbial communities (Kloepper et al. 1980, Haas and Défago 2005, Stilwell et al. 2018, Mirleau et al. 2000, Banin et al. 2005, Sass et al. 2018). The fact that pyoverdin is accumulated and localized under growth limiting conditions in a diverse range of *Pseudomonas*, combined with evidence of the same behavior in SBW25 cells grown for weeks under nutrient restrictive conditions with reliance on cellulose degrading *Bacillus* (Fig. 5), suggests that polarization has relevance to conditions beyond those experienced by cells in standard, nutritionally-rich, exponential-phase, laboratory culture.

Given that pyoverdin accumulation appears connected to cytoplasmic shrinkage, it seems reasonable to assume that other molecules may similarly accumulate in the periplasm in response to division arrest signals, which both change size and shape of cellular compartments. Indeed, accumulation of products related to nutrient stress is one possible function of the bacterial periplasm, with roles analogous to vacuoles in eukaryotes (Brauer et al. 2023).

## Supplementary Material

uqae001_Supplemental_File
