# Supplementary material for: Polar accumulation of pyoverdin and exit from stationary phase
Source: Microlife. 2024 Feb 14;5:uqae001. doi: 10.1093/femsml/uqae001 (PMC10873284; doi:10.1093/femsml/uqae001)
Supplement: uqae001_Supplemental_File [file uqae001_supplemental_file.pdf]

SUPPLEMENTARY INFORMATION: Polar accumulation of pyoverdin and exit from stationary phase

Clara Moreno-Fenoll[1,2,*], Maxime Ardré[1], and Paul B. Rainey[1,2]

[1]Laboratory of Biophysics and Evolution, CBI, ESPCI Paris, Université PSL, CNRS, 75005 Paris, France
[2]Department of Microbial Population Biology, Max Planck Institute for Evolutionary Biology, Plön, Germany
[*]Corresponding author: clara.moreno-fenoll@espci.psl.eu

Supplementary Methods: Image processing and phenotypic classification of cells Images were pre-processed for segmentation using the image analysis software ImageJ [1]. Time-lapses of growing microcolonies were segmented using the SuperSegger Toolbox [2] for Matlab [3]. This toolbox has been designed specifically for the analysis of bacterial microcolonies imaged under phase contrast and fluorescence microscopy and allows the user to adapt segmentation variables to suit a particular experimental system using neural network training. Individual images of cells and their lifetime properties (including time of birth and death, lineage, polar identity, and others) are the ouptut of the software. This enabled tracking of cells throughout three generations mentioned in the main text: F0 or inoculum cells, F1 or exponentially growing cells resulting from the division of F0, and F2 cells or stationary phase cells resulting from the division of F1. Pyoverdin polarization is thus observed in F2 cells. To obtain a means to automatically classify cells by their pyoverdin polarization status the Statistics and Machine Learning Toolbox for Matlab was used [4]. 646 segmented cell images were collected as the training set, originating from 4 different experiments. The training set included images obtained from microcolonies growing on an agarose pad and from cells treated with an iron chelator in liquid medium, to account for differences between experimental setups that could impact on image analysis (e.g. overlapping cells in microcolonies, changing levels of relative cell fluorescence in different environments, etc.) These cells were visually classified into "polarized" and "homogeneous". Next, image features were extracted to develop a classification algorithm. To extract uni-dimensional features from 2-dimensional images, these were processed as follows: first, mean fluorescence along the long cell axis was obtained (also named kymograph). Cells were then divided into three equally sized regions representing the center of the cell and both cell poles and different variables were obtained comparing these regions. Many variables were tested and optimum results were obtained when using the following procedure: kymographs were normalized by either subtracting or dividing by the median fluorescence of the cell center. From these, standard deviation, and 75th percentile of either polar region were calculated. These 6 features were fed into the Classification Learner App in Matlab with 5-fold cross-validation. The model with best results was a linear SVM (Support Vector Machine) model, with accuracy $>$90%. It is estimated that the model has a false negative rate of $\sim$5% for "homogeneous" cells and $\sim$10% for "polarized" cells i.e. it has a slight bias towards under-identification of polarization which we found more desirable than over-identification. This model was used for all subsequent classification mentioned in the main text and SI. To further analyze and visualize data the programming software R was used [5].

Supplementary Methods: Construction of strain with periplasmic red fluorescent marker The IPTG-inducible periplasmic mScarlet reporter was created by first introducing the mScarlet-I CDS from plasmid pMRE-Tn*7*-155 (amplified using primers mScarlet_fwd and mScarlet_rev) into plasmid pUC18-mini-Tn*7*T-LAC (linearized using primers Tn*7*LAC_fwd and Tn*7*LA_rev) using NEBuilder HIFI DNA Assembly Master Mix (New England BioLabs)[6, 7]. The DsbA signal sequence from SBW25 (the first 81 nucleotides of PFLU0083) was then introduced to the start of mScarlet-I in the resulting plasmid by FastCloning [8] using primers dsbAmScarlet_fwd and dsbAmScarlet_rev. The forward primer included the LEGPAGL amino acid linker used by Uehara *et al* (2009) [9] to construct $dsbA_{ss}$-mCherry in *E. coli*. This final plasmid was introduced into SBW25 by electroporation (mediated by the transposition helper plasmid pUX-BF13 [10]) and integration of the transposable element containing Plac-$dsbA_{ss}$-mScarlet-I into the attTn*7* site downstream of *glmS* was selected by plating to gentamicin. Correct integration of the Tn*7* cassette was confirmed by Sanger sequencing. All cloning was performed in *E. coli* OneShot TOP10 chemically competent cells (ThermoFisher Scientific) and PCRs were conducted using either Phusion High-Fidelity PCR Master Mix (ThermoFisher Scientific) or Q5 High-Fidelity 2X Master Mix (New England BioLabs). pMRE-Tn*7*-155 was a gift from Mitja Remus-Emsermann (Addgene plasmid # 118569 ; `http://n2t.net/addgene:118569;RRID:Addgene\_118569`). pUC18-mini-Tn*7*T-LAC was a gift from Herbert Schweizer (Addgene plasmid # 64965 ; `http://n2t.net/addgene:64965;RRID:Addgene\_64965`).

Primers (overlaps for assembly or primer extensions in lower case, annealing region in uppercase, dsbAss in italics, LEGPAGL linker in bold)

Primer	sequence
mScarlet_fwd	cggataacaaGTGAGCAAGGGCGAGGCA
mScarlet_rev	ctgtgtgaaaTTACTTACTTGTACAGCTCGTCCATGC
Tn7LAC_fwd	aagtaagtaaTTTCACACAGGAAACAGAATTC
Tn7LAC_rev	ccttgctcacTTGTTATCCGCTCACAATTC
dsbAmScarlet_fwd	*ctgccagcctcttcggcatgaccgcacaagctgccgacgtgccg**cttctcgagggtccggctggtctg*** atgGTGAGCAAGGGCGAGGC
dsbAmScarlet_rev	*ccgaagaggctggcagtgacgagagcggcgctgaggatcagattacgcatggtctgtttcctgtgtgaaa* TTGTTATCCGCTCACAATTCCACAC

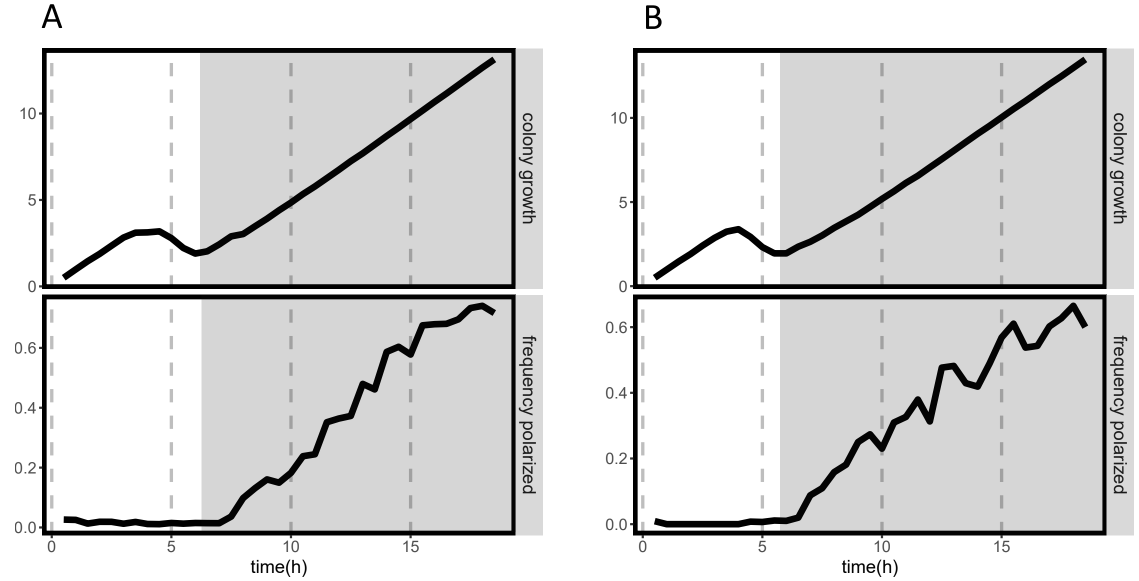

Figure S1: **SBW25 consistently polarizes pyoverdin upon entry into stationary phase**. Polarization in different growth stages of a microcolony. Both plots represent mean age of the cells in a growing microcolony of SBW25 (black line, top) and the corresponding frequency of polarized cells for each time point (black line, bottom). Bacterial colonies undergo different growth stages, from left to right: lag phase (generation F0), exponential phase (generation F1), and stationary phase (generation F2) Colored panels highlight stationary phase of bacterial colony growth, where pyoverdin is observed to accumulate at cell poles. In all cases data has been filtered to exclude cells with segmentation errors or other artifacts that preclude proper analysis. Each plot depicts a biological replicate of the experiment described in Fig. 2A. A) 5 technical replicates, N = 152 initial cells. B) 4 technical replicates, N = 109 initial cells

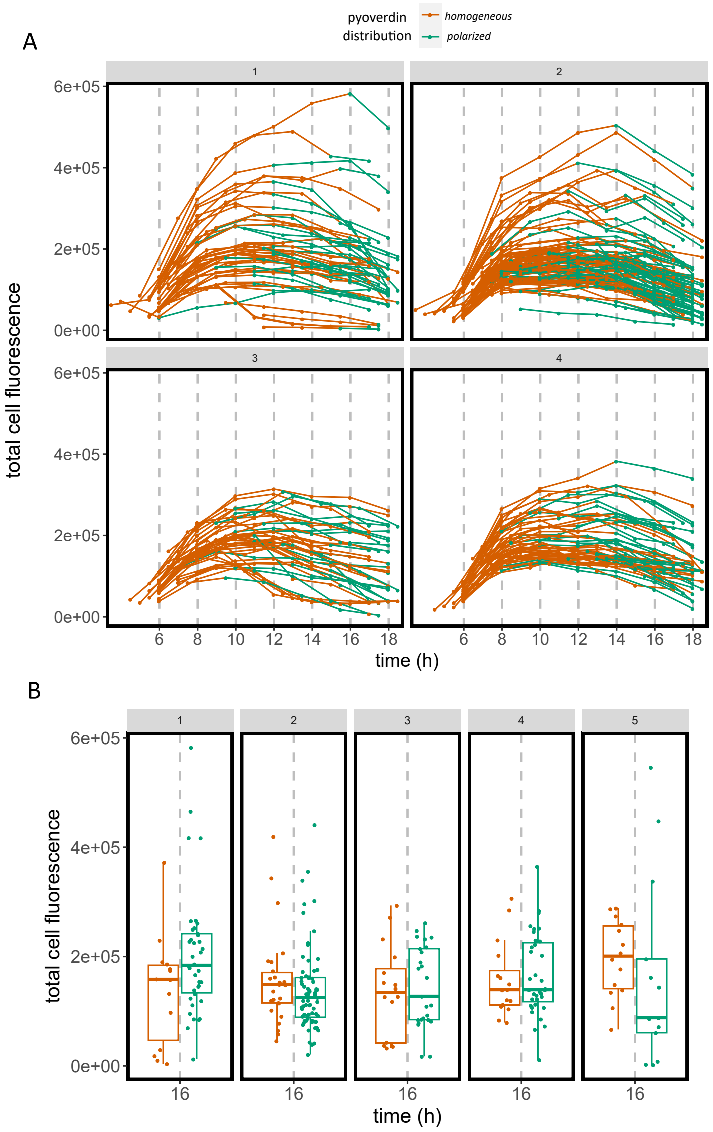

Figure S2: **Total cell fluorescence over time in cells that accumulate pyoverdin at the pole during stationary phase** A) Each line represents an individual cell that is generated by cell division but does not subsequently divide over the course of the time-lapse, i.e. generation F2 as defined in the main text. Values are the sum of the fluorescence intensity of all pixels corresponding to a segmented cell. Lines are colored according to the pyoverdin distribution inside the cell at each time point, classified by a machine learning algorithm in "homogeneous" (red lines) or "polarized"(green line). Each panel labelled 1-4 represents a technical replicate, i.e. a different position in the microscope slide during image acquisition. B) Distribution of total cell fluorescence in cells that are classified as "homogeneous" (red) or "polarized" (green) at a time point corresponding to mid to late stationary phase, t = 16h. Box plots represent the mean and associated statistical parameters. Dots represent individual cell values. Panels represent technical replicates as in A.

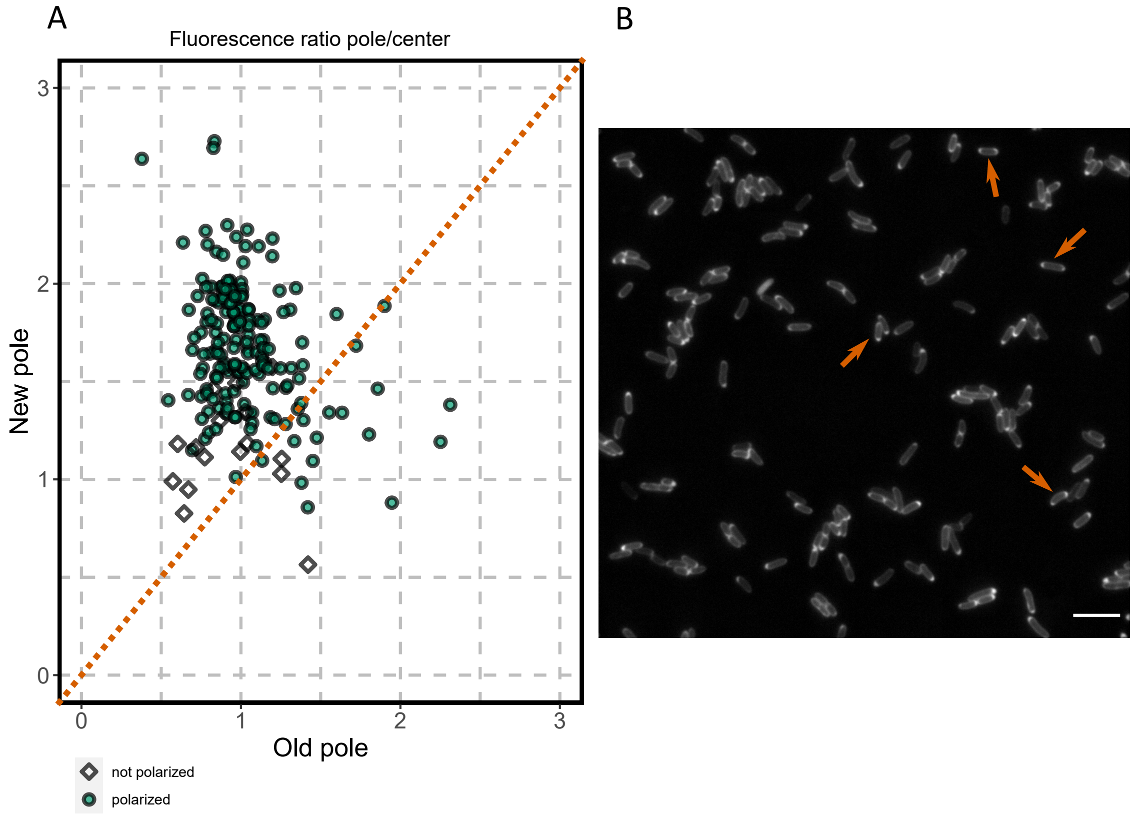

Figure S3: **Accumulation of pyoverdin occurs predominantly at the new cell pole, but not exclusively and occasionally at both cell poles.** A) Ratio of fluorescence between the central region of cells and the region of the new cell pole or old cell pole. Fluorescence values are obtained by dividing segmented cells into 3 proportional regions over long cell axis and averaging fluorescence along both cellular dimensions. Data corresponds to individual F2 cells at time = 18 h, N = 187. Green circles represent cells classified as "polarized", white diamonds represent cells classified as "not polarized". Red line highlights 1:1 ratio of fluorescence in cell centers to cell poles for reference. B) Image of SBW25 cells in conditions that promote polarization, in this case, high cellular density leading to rapid entry into stationary phase (t = 18 h) where accumulation of pyoverdin at both cell poles can be observed in few individuals (red arrows). Scale bar = 10 μm.

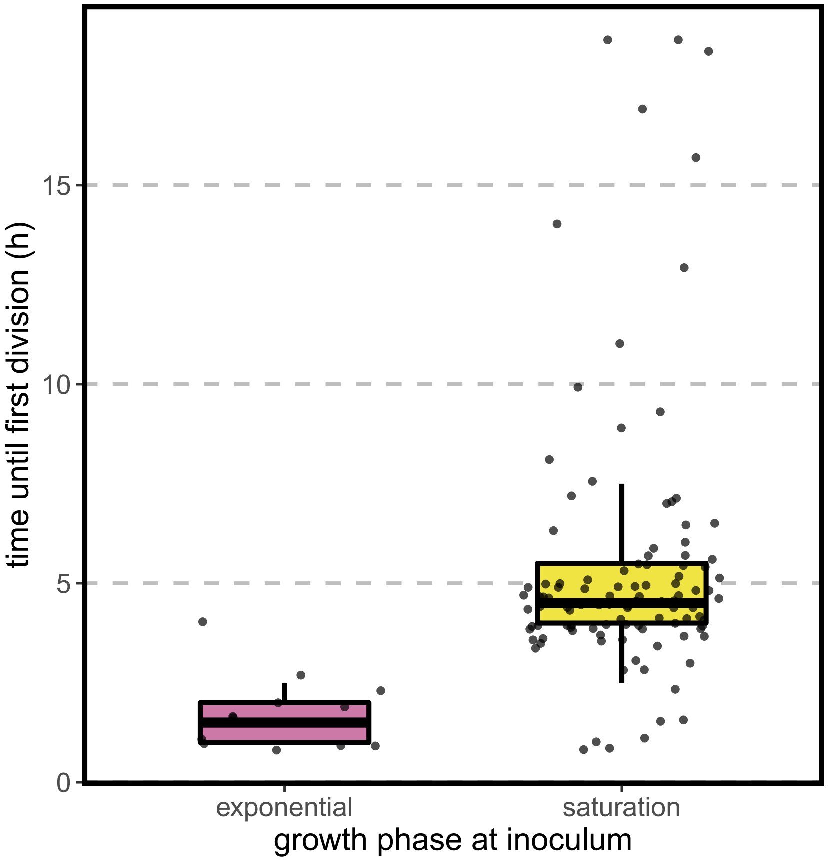

Figure S4: **Lag time difference between cells in stationary and exponential phase** To assess the physiological state of cells after 24 h in SMM (whether they are still in exponential phase due to the minimality of the medium or rather they are entering stationary phase) we compared the time until first division of cells previously resuspended in fresh liquid SMM culture for 4h with data from cells inoculated in a fresh agarose pad directly following the usual procotol described in the main text. In the former case the cells are in exponential phase as their lag time is comparable to division time in this medium. (N = 13, 109 respectively)

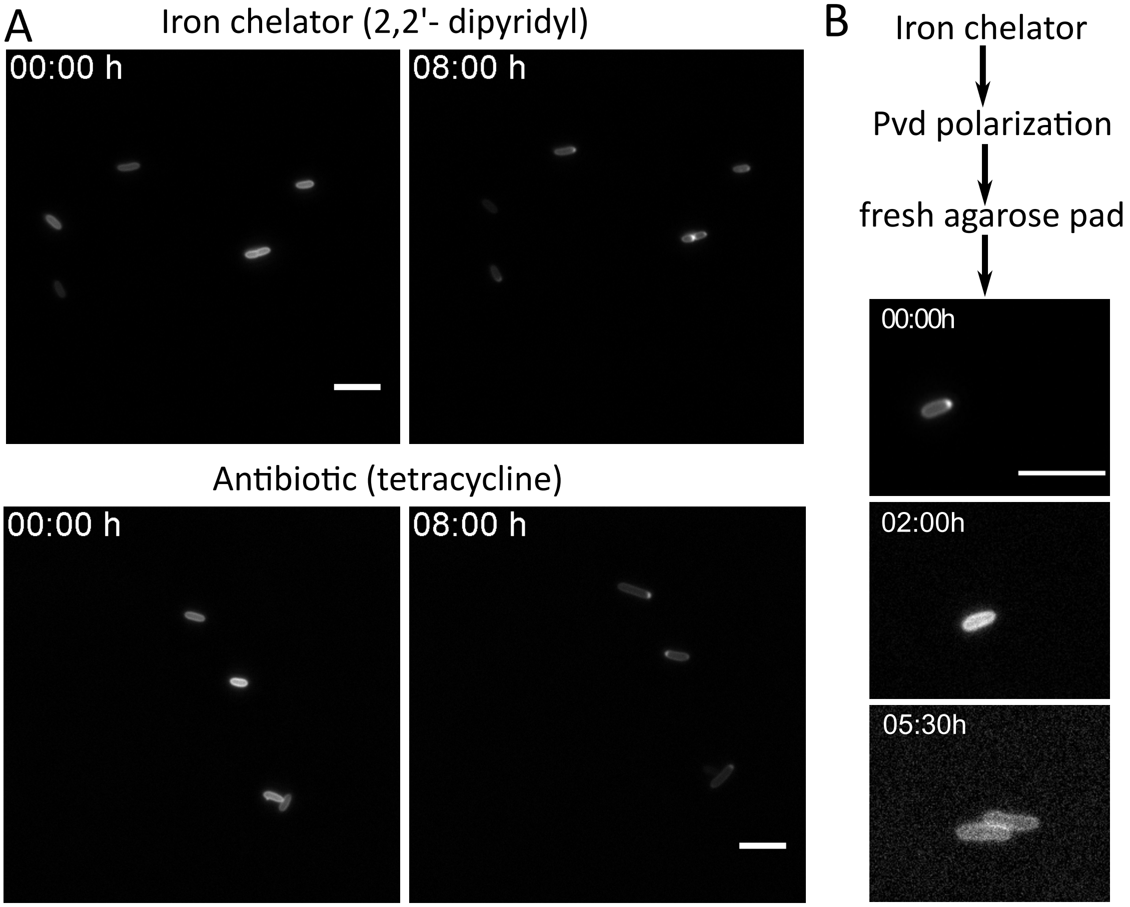

Figure S5: **Exposure to chemicals that prevent cell growth induces reversible accumulation of pyoverdin at the cell pole.** A)Treatment with iron chelator (top) or antibiotic (bottom) triggers accumulation of pyoverdin at the cell pole without detectable cell growth. SBW25 cells were inoculated on an agarose pad containing either 100 μg/ml of 2,2'-dipyridyl (top) or 5 μg/ml tetracycline (bottom). Images were taken at t = 0 h and t = 8 h at the same position on the agarose pad. Note then that the photos depict the same individual cells at both time points, as no cell division was observed. B) Pyoverdin polarization is reversible and precedes recovery of growth after stress. Time-lapse images of SBW25 pre-treated with an iron chelator to induce pyoverdin polarization, then washed and inoculated on a fresh agarose pad. Images follow one individual cell as it recovers homogeneous pyoverdin distribution in the periplasm, followed by cell division.

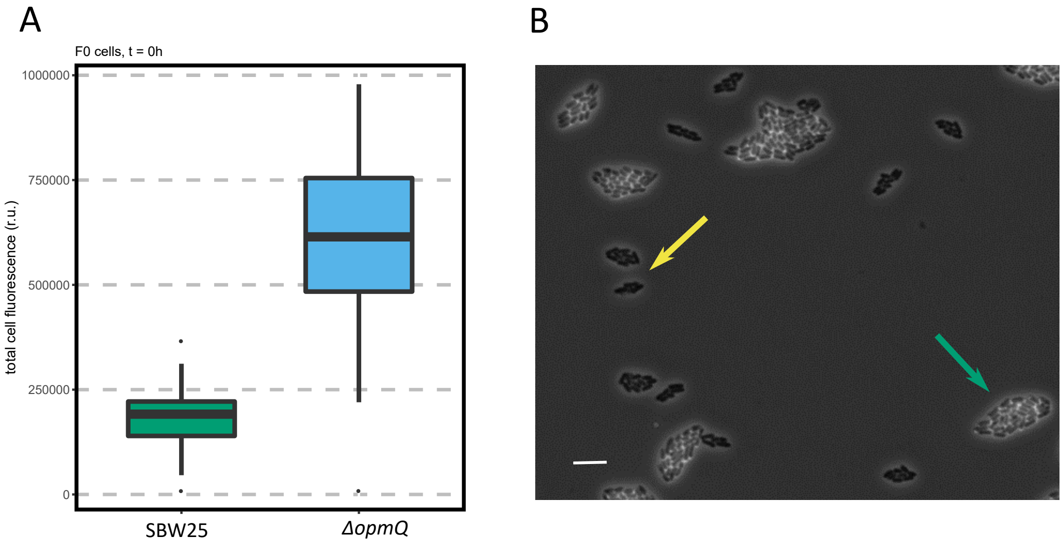

Figure S6: **OpmQ and FpvA deletion mutants are impacted on their ability to recycle, import, and synthesize pyoverdin.** A) Deletion of the export pump OpmQ leads to increased intracellular levels of pyoverdin compared to ancestral SBW25. Box plots represent the aggregated fluorescence of individual cells of SBW25 (green) and $\Delta opmQ$ (light blue) at t = 0 h of inoculation on the agarose pad, N= 35, 29, respectively. B) Deletion of the receptor FpvA reduces pyoverdin synthesis to basal levels, and impedes its import by crossfeeding. Image depicts co-culture of ancestral SBW25 (tagged with mCherry for identification) and $\Delta fpvA$, where phase contrast and fluorescent imaging of pyoverdin have been overlayed to highlight the differences in pyoverdin production between both strains. Scale bar = 10 μm. Strains were mixed in equal proportions and grown on an agarose pad for 18 h. Green arrows point to microcolonies of SBW25, yellow arrows point to $\Delta fpvA$ microcolonies, as representative examples of the two phenotypes.

A

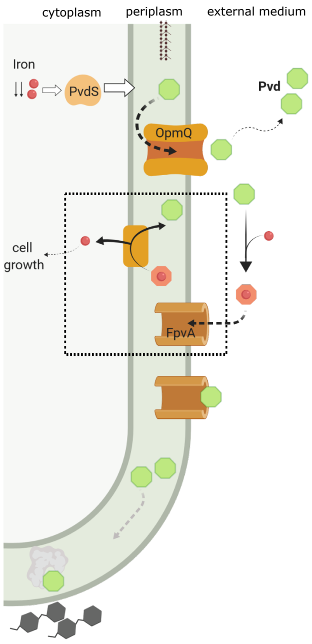

B

FpvA
FpvF
FpvD
FpvG

C

function	PAO1 gene	mutation	phenotype
**reductase**	*fpvG*	*ΔPFLU_2048*	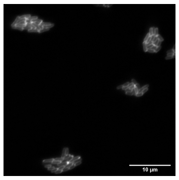
**siderophore binding**	*fpvF*	*ΔPFLU_2041*	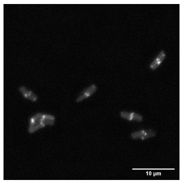
**ABC ATPase**	*fpvD*	*ΔPFLU_2043*	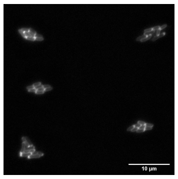

Figure S7: **title** A) Cartoon depicting the pyoverdin pathway, see main text for details. B) Cartoon detailing the mechanism of iron extraction from the ferripyoverdin complex and recycling of pyoverdin molecules. C) Pyoverdin polarization phenotype of a collection of deletion mutants of the processes depicted in B). Genes were selected based on recent work on *P. aeruginosa* PAO1 identifying the function of previously unknown proteins. Orthologs of these genes were identified in *P. fluorescens* SBW25 in the database pseudomonas.com. Genes were deleted by two-step allelic exchange and cells were cultured and imaged as previously described.

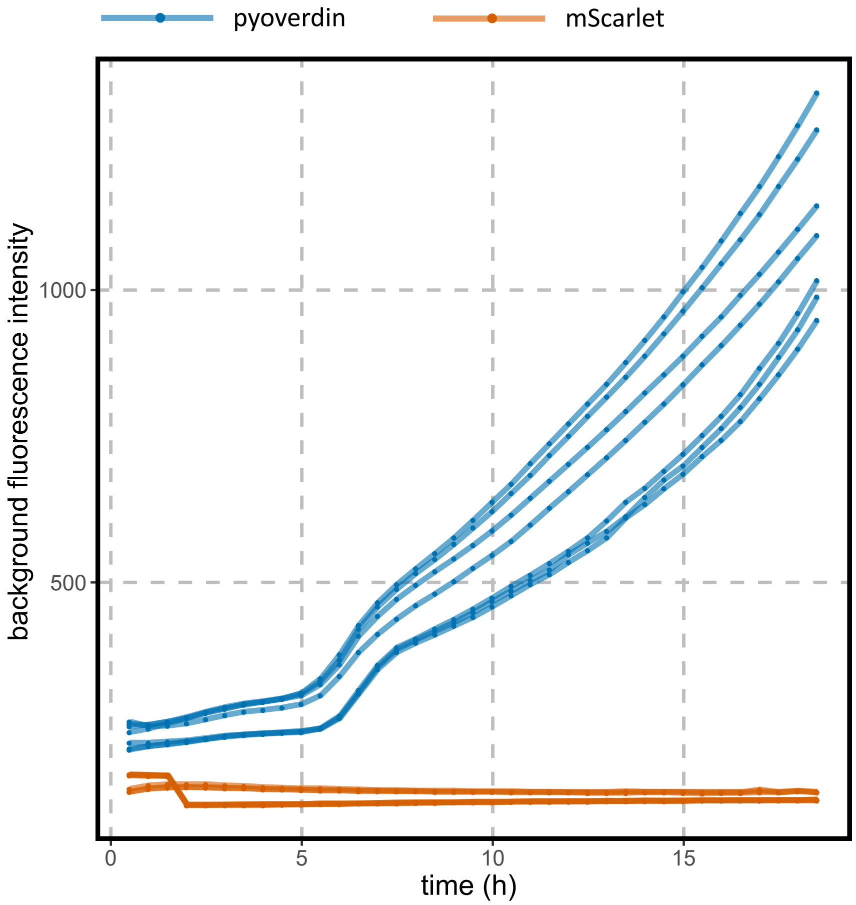

Figure S8: **Fluorescence background measured when imaging cells that produce the naturally-occurring molecule pyoverdin and an engineered periplasmic mCherry protein** Dots and lines represent the median intensity value of a 100x100 px square in the microscope field where cells are not present. Red lines correspond to the red fluorescent protein mCherry while blue lines correspond to pyoverdin (which exhibits a fluorescence emission peak at 450nm). Each line represents a technical replicate.

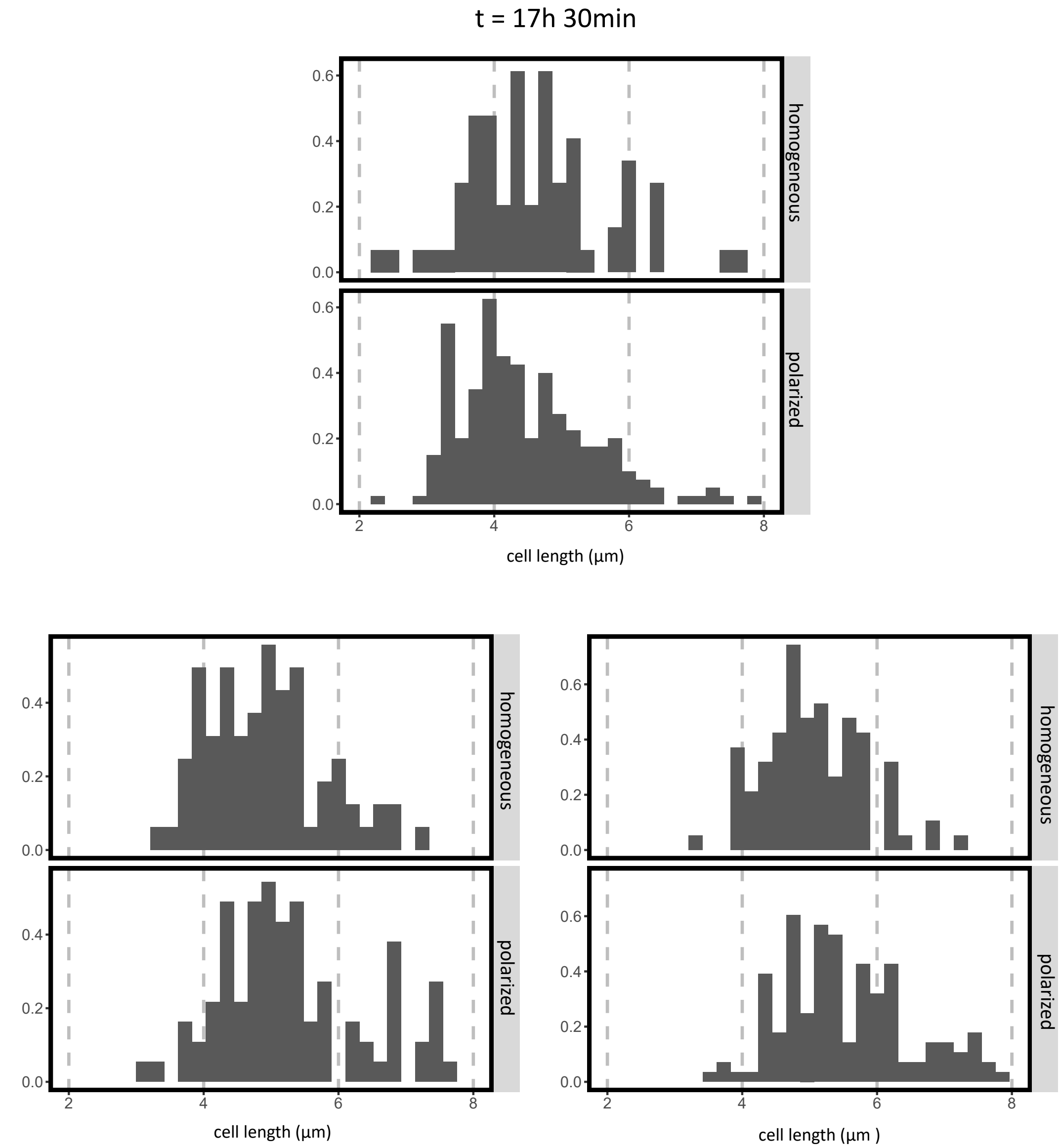

Figure S9: **Accumulation of pyoverdin as a function of cell size.** Distribution of cell size after 17h 30 min of growth in the agarose pad. The population of cells is classified according to the localization of pyoverdin in "accumulated"and "non-accumulated". Cell size is measured as length on the long axis, since elongation occurs in this direction and thus will determine final cell area. Bars represent the distribution of cell lengths in both groups of cells. Each plot represents an independent experiment. Strains used: top: SBW25, bottom: MPB25340 (SBW25 with periplasmic red fluorescent marker), left and right panels represent 2 biological replicates. N = 261 , 167, 235 respectively.

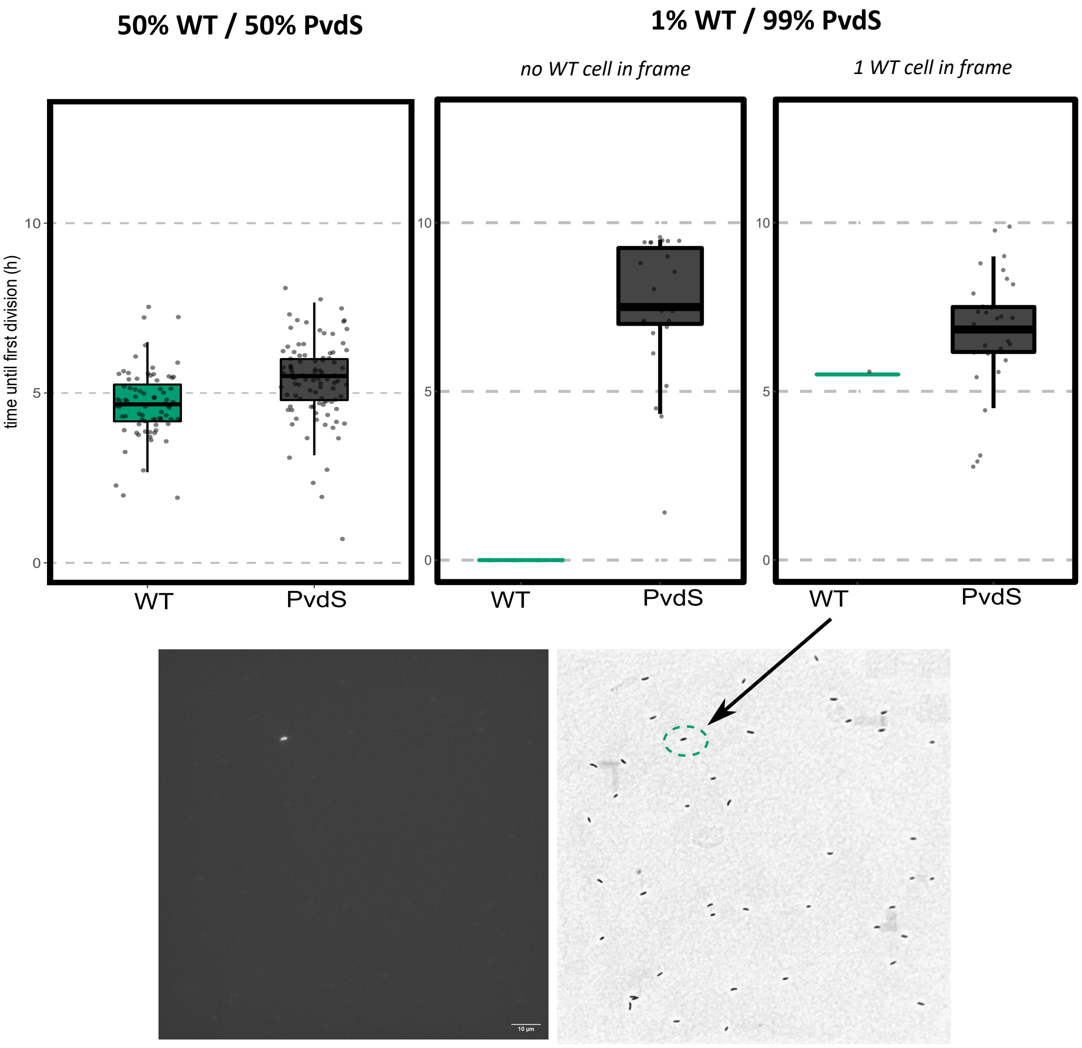

Figure S10: **Lag time of ancestor and nonproducing mutant in co-culture where pyoverdin producer is rare** Time after first division of ancestor and mutant cells pre-treated with DP upon co-inoculation on a fresh agarose pad. Prior to inoculation cells were mixed at 1:1 proportions (left panel, figure is the same as fig 4 B in main text and is included for reference) or ancestor cells at 1%. Center panel depicts data from a technical replicate where no ancestor cells can be identified in the microscope field of acquisition, right panel depicts data from a technical replicate where a single ancestor cell can be identified. Images below correspond to this latter dataset, where left panel represents fluorescent pyoverdin imaging and right panel represents phase contrast imaging. Size of the field of view is 132.06 x 132.06 μm and scale bar represents 10μm The single pyoverdin producer is highlighted in green dashed line.(N = 27, 35 respectively)

References

1. Schindelin, J. *et al.* Fiji: an open-source platform for biological-image analysis. *Nature Methods 2012 9:7* **9,** 676–682 (2012).
2. Stylianidou, S., Brennan, C., Nissen, S. B., Kuwada, N. J. & Wiggins, P. A. SuperSegger: robust image segmentation, analysis and lineage tracking of bacterial cells. *Molecular Microbiology* **102,** 690–700 (2016).
3. Inc., T. M. *MATLAB version: 9.5.0 (R2018b)* Natick, Massachusetts, United States, 2018.
4. Inc., T. M. *Statistics and Machine Learning version: 9.4 (R1028b)* Natick, Massachusetts, United States, 2018.
5. R Core Team. *R: A Language and Environment for Statistical Computing* R Foundation for Statistical Computing (Vienna, Austria, 2020).
6. Choi, K. H. *et al.* A Tn7-based broad-range bacterial cloning and expression system. *Nature methods* **2,** 443–448 (2005).
7. Schlechter, R. O. *et al.* Chromatic bacteria – A broad host-range plasmid and chromosomal insertion toolbox for fluorescent protein expression in bacteria. *Frontiers in Microbiology* **9,** 423623 (2018).
8. Li, C. *et al.* FastCloning: A highly simplified, purification-free, sequence- and ligation-independent PCR cloning method. *BMC Biotechnology* **11,** 1–10 (2011).
9. Uehara, T., Dinh, T. & Bernhardt, T. G. LytM-domain factors are required for daughter cell separation and rapid ampicillin-induced lysis in Escherichia coli. *Journal of bacteriology* **191,** 5094–5107 (2009).
10. Bao, Y., Lies, D. P., Fu, H. & Roberts, G. P. An improved Tn7-based system for the single-copy insertion of cloned genes into chromosomes of gram-negative bacteria. *Gene* **109,** 167–168 (1991).
